# Designing Physical Human-Robot Interaction Interfaces: A Scalable Method for Simulation Based Design

**DOI:** 10.3389/fnbot.2021.727534

**Published:** 2022-02-18

**Authors:** Rohit John Varghese, Gaurav Mukherjee, Ashish Deshpande

**Affiliations:** ^1^Department of Mechanical Engineering, University of Texas at Austin, Austin, TX, United States; ^2^Harmonic Bionics Inc., Austin, TX, United States; ^3^Department Mechanical Engineering, University of Washington, Seattle, WA, United States

**Keywords:** simulation, wearable, robot, haptics, comfort, hand, design methodology, soft tissue artefact

## Abstract

Designing the physical coupling between the human body and the wearable robot is a challenging endeavor. The typical approach of tightening the wearable robot against the body, and softening the interface materials does not work well. It makes the task of simultaneously improving comfort, and anchoring the robot to the body at the physical human robot interaction interface (PHRII), difficult. Characterizing this behavior experimentally with sensors at the interface is challenging due to the soft-soft interactions between the PHRII materials and the human tissue. Therefore, modeling the interaction between the wearable robot and the hand is a necessary step to improve design. In this paper, we introduce a methodology to systematically improve the design of the PHRII by combining experimentally measured characteristics of the biological tissue with a novel dynamic modeling tool. Using a novel and scalable simulation framework, HuRoSim, we quantified the interaction between the human hand and an exoskeleton. In the first of our experiments, we use HuRoSim to predict complex interactions between the hand and the coupled exoskeleton. In our second experiment, we then demonstrate how HuRoSim can be coupled with experimental measurements of the stiffness of the dorsal surface of the hand to optimize the design of the PHRII. This approach of data-driven modeling of the interaction between the body and a wearable robot, such as a hand exoskeleton, can be generalized to other forms of wearable devices as well, demonstrating a scalable and systematic method for improving the design of the PHRII for future devices coupled to the body.

## 1. Introduction

**Figure 1:**
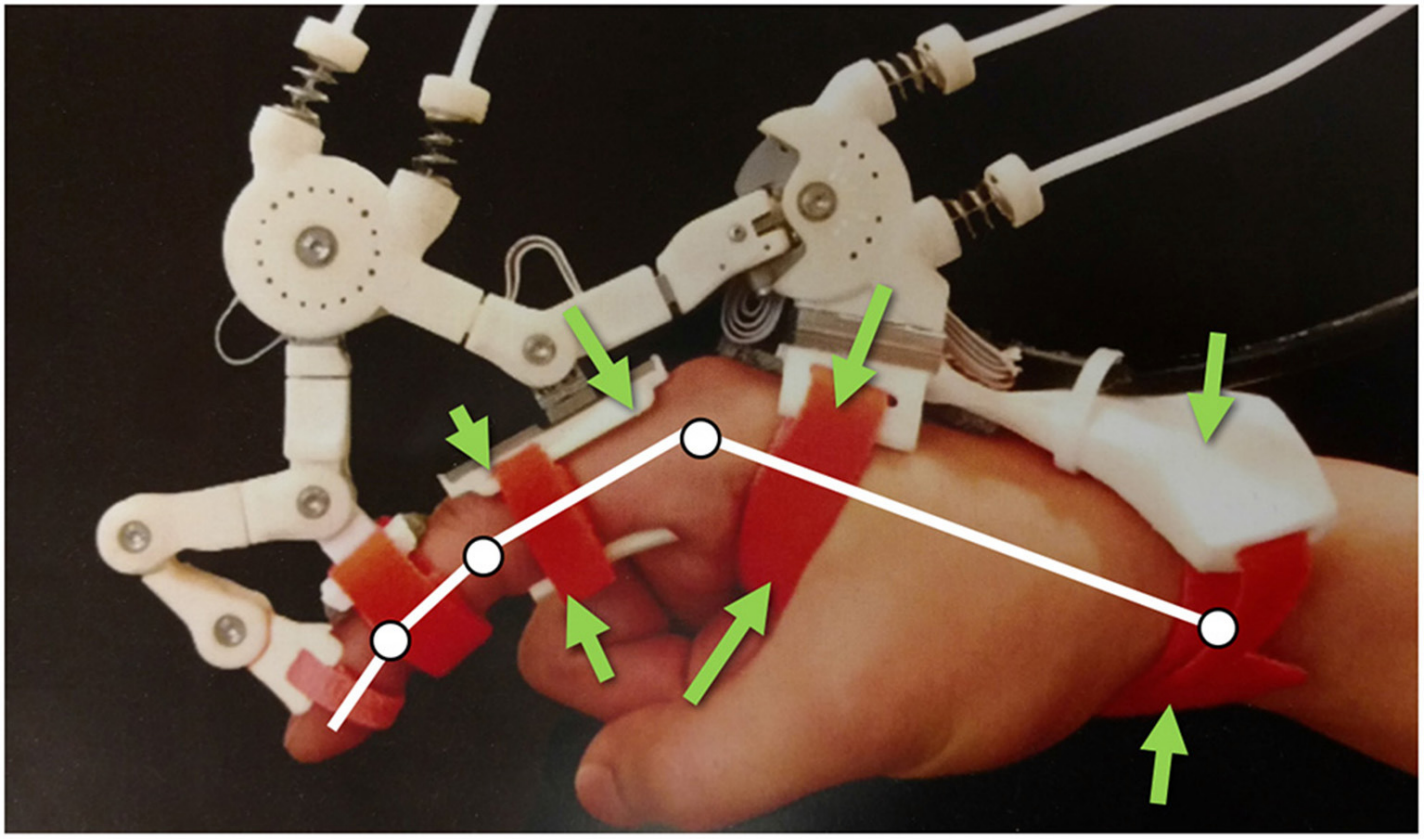
Maestro Hand Exoskeleton from Agarwal et al. (2015), with force arrows indicating various locations of PHRII in this system.

Robots coupled to the human body assist in the rehabilitation of impaired mobility (Gupta and O'Malley, 2006; Kim and Deshpande, 2017; Yun et al., 2017), provide assistance to humans in performing tasks (Bogue, 2009; Walsh et al., 2016), and augment physical interactions in virtual and augmented reality (Choi et al., 2016; Pezent et al., 2019; Young and Kuchenbecker, 2019). These robotic systems function by transmitting forces across the physical interface between the robot and the human. Often referred to as the Physical Human Robot Interaction Interface (PHRII) Figure 1, these attachment locations are complex sub-systems that involve multiple layers of tissue (skin, fascia, sub-cutaneous tissue, muscle, etc.) along with compliant padding and attachment straps of the device.

When forces are applied across the stack of PHRII and tissues, complex static and dynamic interactions occur, resulting in phenomenon, such as slip (Akiyama et al., 2016), localized pressure (Krouskop et al., 1985), and lift-off of the robot from the skin. These complex interactions cause significant uncertainty in estimating the relative position and force distribution between the robot and the human. These uncertainties adversely impact the control framework of the robot, as well as the ergonomics and safety considerations for the human. For example, minor relative displacements, or slip, can cause misalignment between the joint axes of the human and robot (Schiele and Van Der Helm, 2006) resulting in high undesired forces on the human joints (Colombo et al., 2000). These relative displacements also cause errors in the control model of the coupled human-robot system which can lead to instability, especially when using model-sensitive strategies such as adaptive control (Dubowsky and Desforges, 2015). Currently, the behavior of the PHRII is not well understood, which limits our ability to design and control the interaction between the human and the wearable robot. Therefore, to optimize the PHRII, we must first characterize the behavior at this interface. To achieve this goal, we present a novel data-driven approach of characterizing the tissue properties locally to model and optimize the global design of the PHRII.

While an overall model for the PHRII doesn't exist in the literature, prior studies have addressed some issues related to PHRII design and robot control: (Schiele and Van Der Helm, 2006) looked at ensuring concentricity of the joint axes of the coupled human-robot system by using extra degrees of freedom in the exoskeleton for passive realignment. Agarwal et al. (2015) showed that the use of redundant measurements on joint angles followed by optimization techniques could be used as estimators of the unknown positions of the human in control. Petron (2016) proposed that the most effective method of force transfer across prosthetic leg socket while minimizing discomfort and tissue damage was to tune the stiffness map of the socket to ensure equalized pressure distribution at all points of contact. However, this approach has not been tested for the upper limb. Quinlivan et al. (2015) experimentally showed the benefit of inverting the stiffness profile of he human hip to design the attachment, which is in contact with superficial skeletal structures than surfaces resting against softer tissue. Most studies in this area are recent, and the design of the PHRII is most commonly performed through an iterative, prototype-based process (Bouzit et al., 2002; Kim and Deshpande, 2009). Designers iterate through options for parameters such as compliance of the PHRII for their specific device (Silver et al., 2001), and use features such as the positional errors between the human and robot (Cempini et al., 2014) to measure the effectiveness of the PHRII. This approach is time consuming, expensive, and is difficult to standardize.

To standardize the approach of designing the PHRII, we must first characterize the interaction between the human and the coupled wearable robot. To do this, we developed a novel simulation-based model of the PHRII in MATLAB (Mathworks Inc., MA). This model, called HuRoSim, includes the viscoelastic properties of the human skin and soft tissue, along with an interface material, and attachment straps of the device. HuRoSim focuses on relative movement between the human and the robot, and forces generated at the PHRII as a result of applying loads across it. This approach can be used to compute optimal values of the design parameters, such as geometry, compliance, strap stiffness, pre-tension, etc., quickly by systematically varying them in simulation. It also enables the analysis of robot configurations and the effects of variations in human size and tissue properties.

**Figure 2:**
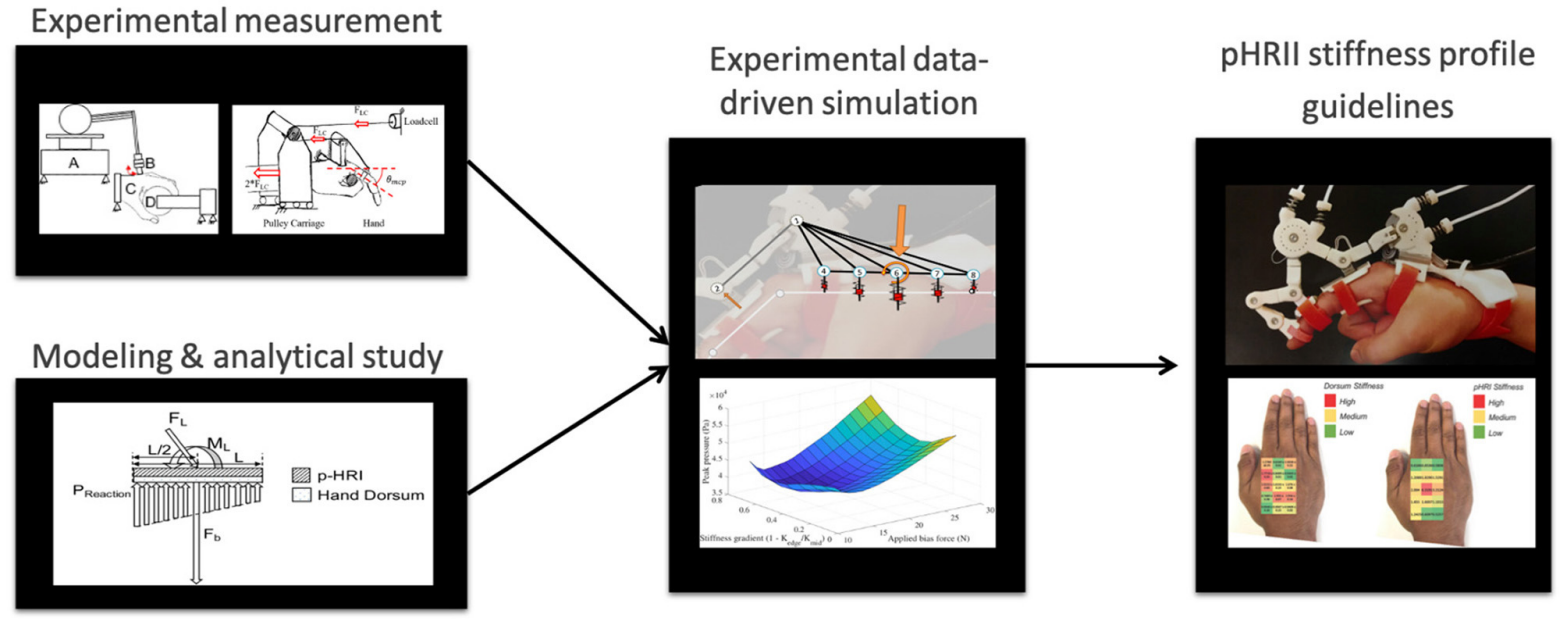
A schematic of our methodology for optimizing the PHRII for the hand dorsum using a data-driven simulation approach.

In the sections that follow, we first introduce our novel simulational framework, HuRoSim followed by two experiments to outline our novel method of optimizing the PHRII (Figure 2). In our first experiment, we design an isometric loading condition for the simulation environment and predict the kinematic behavior of an exoskeleton attached to the hand, under an externally applied load. We then test the predictions from HuRoSim with an experimental set-up. In the second experiment, we demonstrate how the measurement of parameters of the biological tissue, such as dorsum stiffness of the hand, can be used to design a PHRII to optimize comfort.

## 2. Materials and Methods

### 2.1. HuRoSim: The PHRII Simulation Environment

HuRoSim is a dynamic simulation framework built in Matlab 2016 (Mathworks, MA), to characterize the interaction behavior at the PHRII between the hand exoskeleton and the hand. HuRoSim is built as a lumped parameter system, arranged as a network of nodes in 3D space. These nodes are connected by constraints, each with its own set of parameters and properties. This modularity allows the user of HuRoSim the ability to reconfigure the nodes, and simulate a variety of PHRII systems. The user is also able to choose the complexity of the simulational representation of the PHRII, thus trading between computational load and accuracy.

Our simulation model of the PHRII includes a number of features that can affect the interaction between the rigid reference structure of the robot and the human reference (skeletal) structure. This includes: viscoelastic properties of the human tissue (skin, fascia, and subcutaneous tissue) PHRII dimensions and geometry, mechanical properties of the padding used at the interface, slip along the interface, and the mechanical properties including the initial force between the device and tissues or pre-tension of attachment straps.

#### 2.1.1. Viscoelastic Properties in HuRoSim

**Figure 3:**
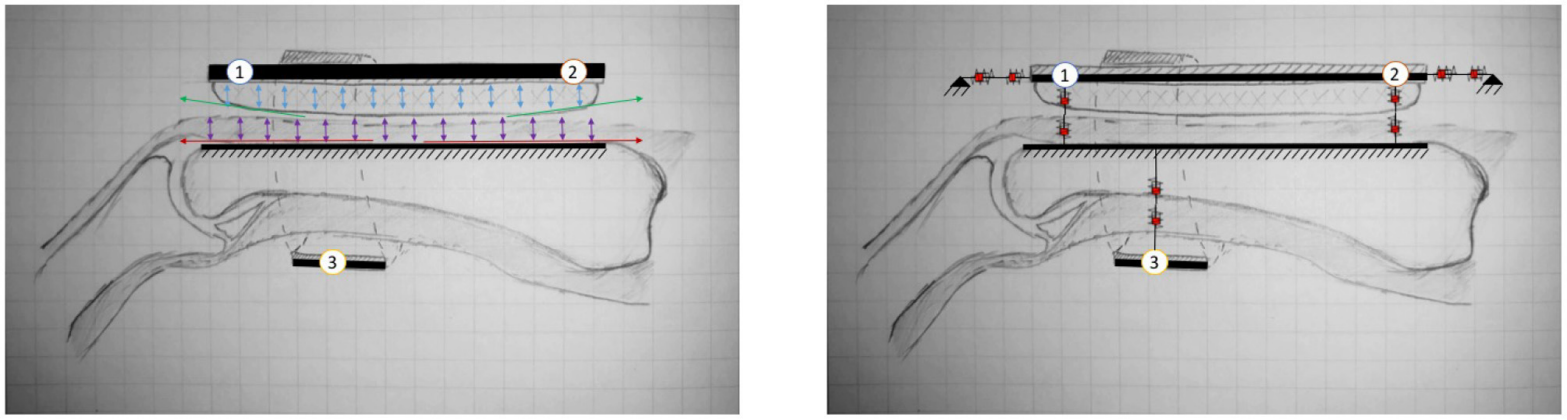
**(Left)** A cross section view of a typical PHRII showing the human reference structure as the ground (the proximal phalanx of the index finger in this case) and the rigid reference structures of the robot above and below it. The numbered circles represent the PHRII units that this structure will be simplified to later in the analysis. **(Right)** A typical PHRII with a schematic representation of the viscoelastic elements. Each simulated point is considered as two elements coupled in series, representing the human soft tissue and the device's compliant padding, respectively.

Figure 3 **(Left)** shows a schematic cross section view of a typical PHRII. The human reference structure (the skeletal bone) is shown in the middle, completely encased by soft tissue both above and below it. The bone in this figure is the proximal phalanx of the index finger, and it's dorsal surface (above the bone in this figure) can be approximated to a straight line. Over the bone is a layer of soft tissue and skin represented by arrows showing its mechanical compliance in compression and sliding. Above the finger is the rigid plate of the robot attachment represented by a solid line, and its compliant padding in contact with the skin on the dorsal surface is similarly represented by arrows showing its mechanical compliance.

The PHRII region is defined by this contact surface described above, and it extends from the rigid reference geometry of the human to the rigid component of the robot. Also visible in Figure 3 **(Left)** is the antagonistic strap that is in contact with the skin on the palmar surface of the hand (below the bone in this figure). Identical to the considerations on the dorsal surface discussed previously, in this PHRII, we also have the two mechanically compliant regions of human soft tissue and device padding between the bone and the inelastic fibers of the strap which we consider as its reference geometry.

The Figure 3 **(Left)** also shows individual sources of movement as colored arrows. The strain distribution in the human soft tissue and the device padding are shown in purple and blue, respectively. Shear strain in the padding is also accounted for in our model, but not explicitly highlighted here. The human skin can also glide over the underlying layers of fascia, as shown in red. The reaction forces to this movement come from two main sources: stretching of the skin itself, which is dominated by the viscoelastic properties of the dermis, and the connective tissue linking the dermis to the underlying structures in the hypodermis and fascia. Lastly, we also have surface effects of slip and lift-off at the actual interface surface, as shown in green.

#### 2.1.2. The Individual PHRII Unit

All components in the PHRII unit are modeled as viscoelastic elements. We use lumped parameter models to represent the robot and strap structures as linked point masses, and the human bone as the reference ground. With this approach, we represent the soft structures in the PHRII as viscoelastic elements connected in series as shown in Figure 3 **(Right)**. This system can be viewed as being composed of three PHRII units, represented by the numbered circles. Each PHRII unit is connected to the human reference ground through series viscoelastic elements, and to each other either with compliant, or with inelastic constraints.

**Figure 4:**
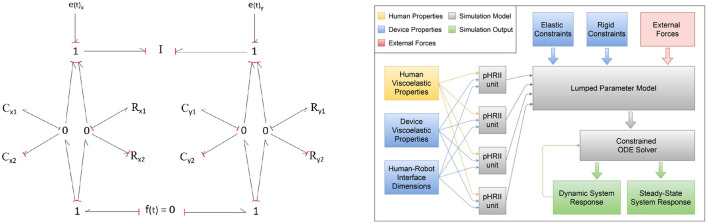
**(Left)** Bond graph of a single PHRII unit. The left half represents features in the tangential or *x*-direction while the right represents normal or the *y*-direction. **(Right)** Block diagram showing information flow across the different elements of the simulation environment.

Figure 4 shows a bond graph representation of a single PHRII unit. The left half represents features in the tangential or *x*-direction while the right represents normal or the *y*-direction. Therefore, *C*_1*y*_ & *C*_2*y*_ represent the compressive elastic properties of the soft tissue and padding, while *C*_1*x*_ & *C*_2*x*_ represent the stretching/sliding of skin over layers of fascia and the padding shear strain. *R*_1*x*_, *R*_1*y*_, *R*_2*x*_, and *R*_2*y*_ represent the viscous properties of each of these elements. The normal and the tangential strains are orthogonal and considered independently. However, they are still connected to the same inertia and reference ground. This individual unit is replicated at every PHRII and is used as the building block for the simulation environment. As seen in Figure 4 **(Right)**, these units are coupled by both elastic and inelastic constraints, as well as external forces and this is the input to the primary lumped parameter model. The lumped parameter model is solved using a constrained ODE solved detailed in the next sub-section. The steady state and dynamic responses of the solver are the outputs which we use for further analysis.

In our example system, the dorsum of the hand has two such PHRII units connected by an inelastic constraint (simulating a rigid body) The palmar surface has a single one which is connected to the dorsal link via another viscoelastic element (simulating the strap). We will be discussing the construction of more complex systems with this building block as we go forward. Note that we have made the assumption of coupling the elastic elements in series separately from the viscous ones. Condition checks were included in the simulation of the system to ensure that this does not create unrealistic behavior in exception cases. These include negative normal forces at the interface on rapid withdrawal of loading.

We use experimentally obtained values for human tissue parameters where available in the literature. These include tensile viscoelastic properties of the skin (Silver et al., 2001; van Kuilenburg et al., 2012; Dabrowska et al., 2016), and compressive viscoelastic properties of the skin and the sub-cutaneous tissue (Wu et al., 2007). The thickness and size of all these regions across population samples are obtained from anthropometric data. The geometry and mechanical properties of the padding and straps can either be selected from a list of commonly used configurations or else optimized by the simulation to be returned as a design input.

#### 2.1.3. Combining Individual PHRII Units to Create a Simulation System

**Figure 5:**
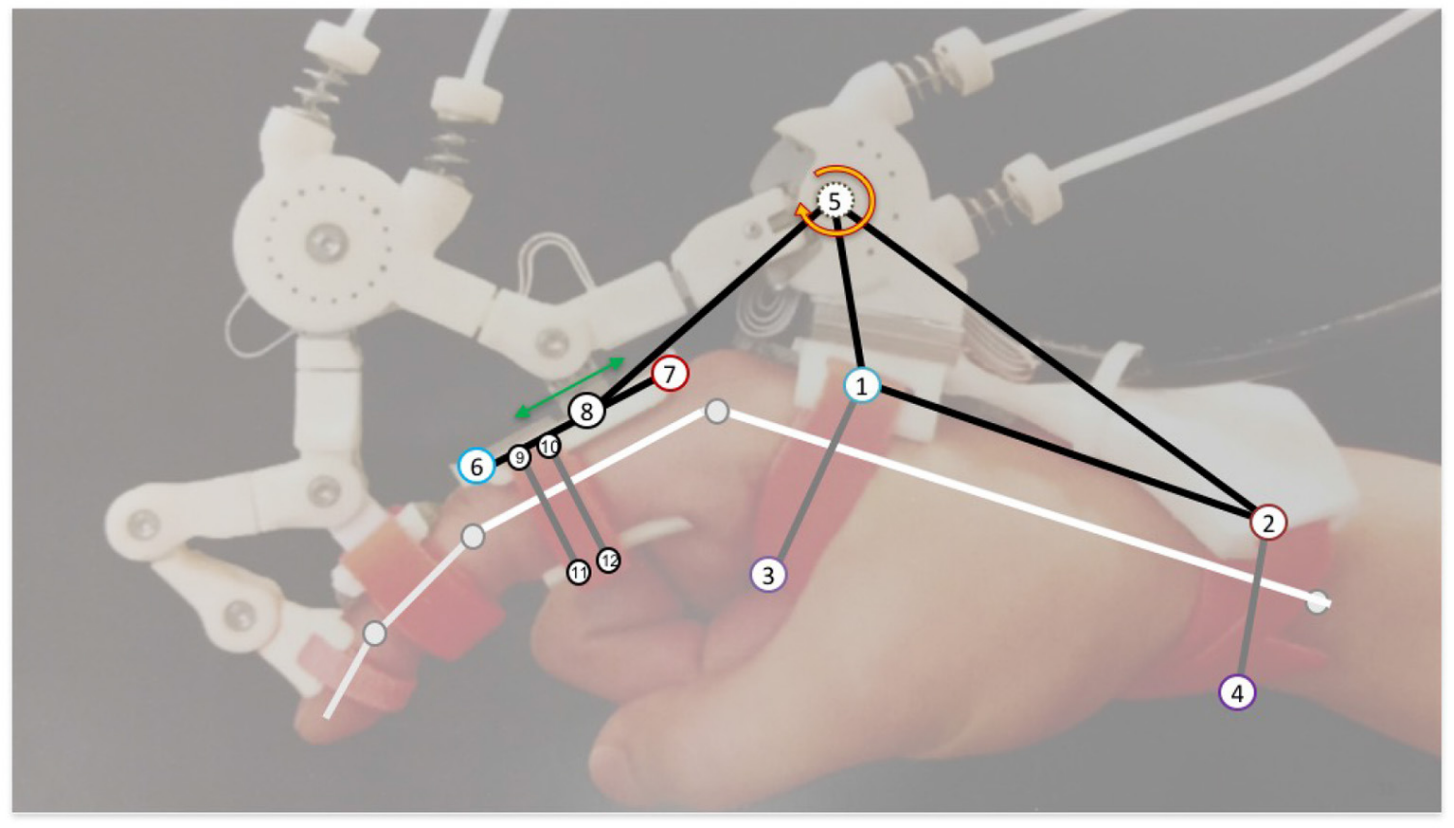
Annotated Maestro system showing the symbolic human reference geometry (white), and the different pieces of the lumped parameter structure used to model and simulate the behavior of this system. Each numbered circle represents a PHRII (Though points 5 and 8 have their viscoelastic values set to zero as described in this section). The black lines represent inelastic constraints while the gray ones represent viscoelastic ones.

Using the PHRII units defined above as our building block, we create larger systems for simulation. Figure 5 shows the system of the Maestro hand exoskeleton with our simulation model superposed over it. We first draw attention to the numbered point masses shown connected by inelastic constraints. Ternary links or sets of three connected masses (such as 1-2-5 and 6-8-7) move together essentially as a rigid body. The gray connections are elastic constraints to simulate compliance of the straps or in the exoskeleton linkage. Note that the human reference structure (shown as white linkages) will not be explicitly visualized in this system going forward, since it is considered as the reference ground. The proximal phalanx is considered as a moving reference ground based on transformations on the human MCP joint. Lastly, not all of the numbered points are in actual contact with the human (for example, points 5 & 8). The PHRII viscoelastic properties for these are set to zero while those for all others are set based on the human and padding viscoelastic properties discussed above.

These lumped parameter models are used in our dynamic simulations, allowing a system approximation that can be tuned to give reasonably accurate results while still being light enough to run in a real-time control loop. Since many of the experimentally determined properties of skin in literature have been approximated to piece-wise linear models (Silver, et al. Wu, et al.), our simulation also uses smoothed piece-wise linear models for tissue viscoelastic properties wherever possible based upon the desired complexity.

#### 2.1.4. Solving the Constrained Multi-Body System Through Time

Dynamic simulations of the resulting multi-body system are formulated with ODE (Ordinary Differential Equation) solvers, and constraints are implemented with the option of simulating in both 2D and 3D using Lagrangian mechanics by Udwadia and Kalaba's method (Udwadia and Kalaba, 2002) as shown in Equation 1 (Here *q*'s are the coordinates of each point, *M* is the overall inertia matrix, *Q* is the array of non-constraint forces for all points, and *A* & *b* are coefficients of the constraint equations after differentiating twice and grouping in the form of Equation 2). This method was chosen over penalty methods and simultaneous solutions for numerical stability and computational simplicity (Bayo and Ledesma, 1996; Witkin and Witkin, 2001).


(1) $$\begin{array}{r} M\ddot{q}=Q+M^{1/2}{\left(AM^{-1/2}\right)}^{†}\left(b-AM^{-1}Q\right) \end{array}$$

(2) $$\begin{array}{r} A\left(\overset{∙}{q},q,t\right)\ddot{q}=b\left(\overset{∙}{q},q,t\right) \end{array}$$

To simulate dynamics through time, the ODE's can be solved both by implicit, as well as explicit methods. Standard ODE solvers are available for both kinds, with each having their respective advantages. Explicit adaptive step Range Kutta solvers such as Matlab's ODE45 are the most common type employed for differential equations. However, with numerically stiff systems, they tend to decrease step size drastically resulting in long computation times. Implicit solvers on the other hand are more resistant to this phenomenon and are capable of computing the dynamics of the system with far fewer temporal steps. However, care must be taken while using them as they are much more prone to numerical instability depending on the system parameters. The simulation environment makes use of both types of solvers to allow for explicit solvers for computational in general cases and for the use of implicit solvers in numerically stiff regions of the system.

The entire simulation was custom built in the Matlab environment (Mathworks Inc.) and provides both steady state and transient responses. However, due to the slow and almost quasi-static movement speed that is used in rehabilitation, assistance, and assessment, our results focused largely on the steady state response.

### 2.2. Experiment 1: Predicting Physical Interaction Using HuRoSim

In this experiment, we study the case of a hand exoskeleton attached to the dorsal aspect of the hand. Using HuRoSim, we apply an extension moment load about the MCP joint to the virtual finger which is fixed at an angle. From this simulation study, we predict the kinematic movement of the coupled hand-exoskeleton system. We then replicate this set-up with the Maestro hand exoskeleton attached to a human hand to measure the actual kinematic effect of applying an isometric load across the MCP joint.

#### 2.2.1. Simulation Experiment

We configured HuRoSim to study the effects of applying an isometric extension load across the MCP joint. The input load to the simulation is in the form of an applied moment from the exoskeleton (to a maximum of 0.5 Nm), and the studied outputs are the final displacements and forces present in the system on reaching a steady state.

The human and exoskeleton interface material parameters were gathered from literature sources, most notably (Silver et al., 2001) which establishes the piece-wise linear approximation of stiffness properties of human skin caused by the behavior of different fibers (elastin and collagen) within the dermis of the skin. In the tangential direction, the values from this source start with an initial elastic stiffness of 0.1 MPa upto a strain of 0.4, followed by a sharp increase trending up to 18.8 MPa. Wu et al. (2007) similarly establishes values of compressive stress-strain curves for both skin and sub-cutaneous tissue that were used in the simulation. It should be noted that detailed *in-vivo* measurements of the tissue properties of the surfaces of the human hand have not been widely studied, leading to a lack of available literature to obtain measurements from. This scarcity contributed to the motivation for indentation experiments in section 2.3.3 that gather *in-vivo* stiffness data of the hand dorsum for use by HuRoSim.

The device parameters were measured directly on the maestro hand exoskeleton, having a dorsal rigid surface of 50 mm length for the metacarpal region and 30 mm for the proximal phalanx of the index finger, with strap widths of 10 mm.

From the applied moment loads to the isometric finger and the tissue properties chosen, we are able to simulate the dynamic loads that develop. These load patterns generate kinematic behavior that we then tested with an experimental set-up with the Maestro hand exoskeleton and the hand.

#### 2.2.2. Experimental Measurement

**Figure 6:**
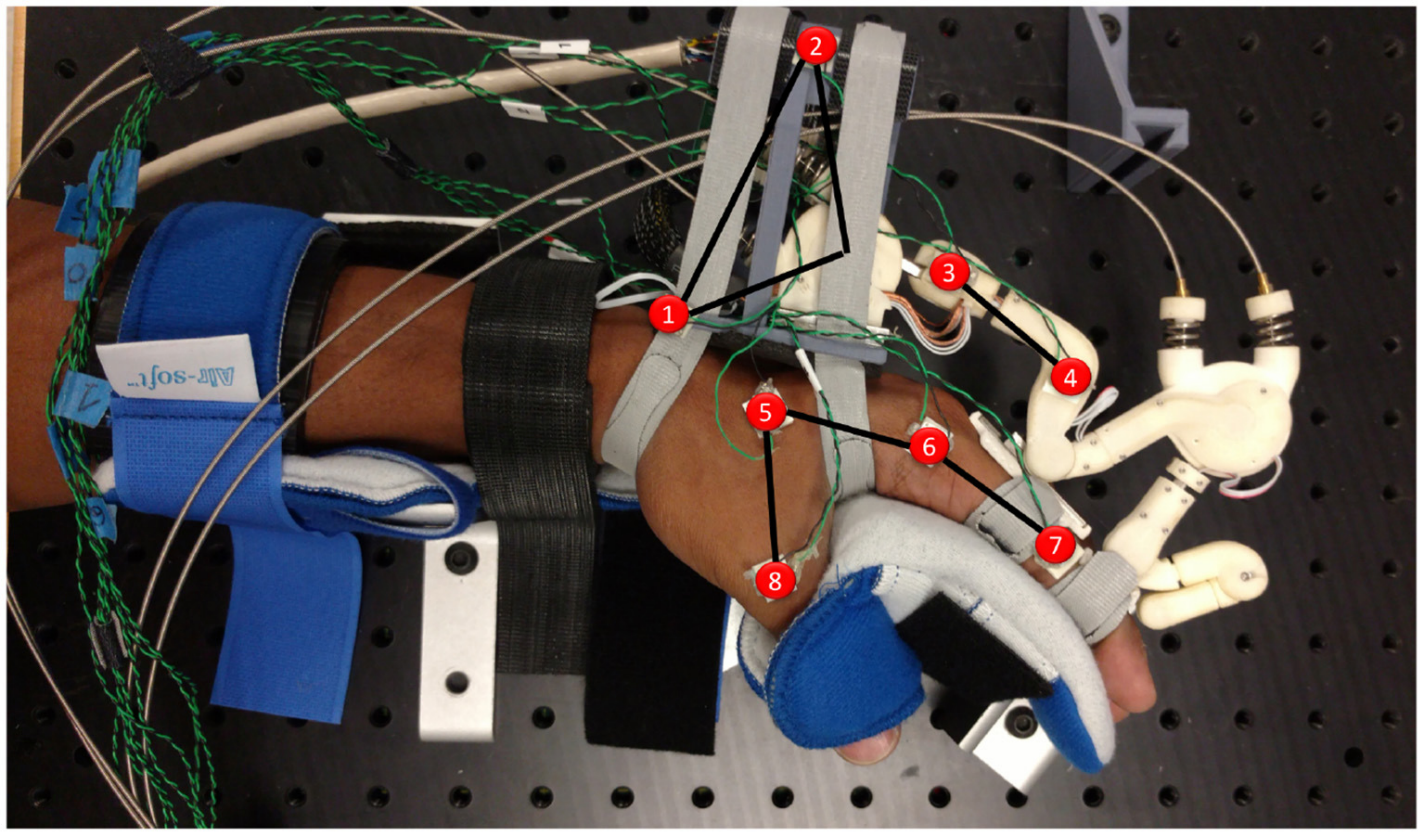
Experimental setup for comparison to the simulated system. The human hand is held in a commercial brace reinforced and bolted down with aluminum channels (seen between the fingers and the thumb on the palmar surface of the hand). The Maestro hand exoskeleton is affixed with a modified base (link 1–2) that allows calibrated pre-tensioning of both straps. The Motion capture sensors are highlighted in red.

We test the predicted kinematic behavior from the simulation with an experimental test bed. We instrument the maestro hand exoskeleton Figure 6 to enable easier measurement of the effects of applying an isometric load across the MCP joint. The index finger was locked about the MCP, PIP, and DIP joints, and an external moment identical to that applied in the simulation was developed over the hand.

Only the index finger of Maestro was attached to the human test subject, and loads were applied via Maestro's MCP joint. The subject's arm is immobilized at the forearm and wrist, in addition to the palmar surfaces of the hand, fingers, and thumb. Phase space X2E motion capture markers were attached to multiple points on key linkages of the maestro exoskeleton. Markers were also placed on the metacarpal, MCP and PIP joints of the index finger and the base of the thumb to track the position of the palm and the index finger. This enables a fair comparison to the simulated system by confirming that the movement of the human hand is minimal.

The required moment load was applied using the Maestro exoskeleton's torque sensing series elastic actuators. To achieve a known level of strap tension, pre-tensioning was carried out using a calibrated Omega DFG55 load cell, rated for 25N. Strap pre-tensioning was performed prior to placing the hand in the brace of the experimental setup by affixing the Maestro grounding link to a rigid fixture through a dovetail joint and aligning the hand within it. The straps were then pulled to the target tension with the load cell attached in series. To ensure minimal effects of friction between the strap and the palm during this step, both ends of all straps were pre-tensioned individually in a consistent order of: distal-lateral, proximal-lateral, distal-medial, proximal-medial. A maximum displayed error of 0.1N was allowed in the experiment protocol during the fastening process.

### 2.3. Experiment 2: Optimizing the Design of the Hand Dorsum PHRII

In addition to characterizing reduced order interactions between lumped parameter representations of interaction, HuRoSim has the ability to provide trends across surfaces of lumped parameter representations to understand the effects of variations in parameters describing neighboring regions. To evaluate this feature of the simulation approach and to demonstrate the value of a simulation tool in the design of the PHRII, we designed an experiment to optimize the design of the PHRII for comfort, and evaluate the new design for performance.

To optimize the PHRII between the hand dorsum and the hand worn exoskeleton for user comfort, we developed a workflow in the HuRoSim simulation framework by defining the spatial relationship between individual lumped mass representations of the points on the hand dorsum and adding in experimentally quantified biological tissue properties (Silver et al., 2001; Wu et al., 2007). At each of the points, the pressure distribution across the PHRII is tracked by HuRoSim as a measure of discomfort. This approach was taken to ensure that an overall picture of the distribution of pressures could be derived to inform an optimization framework.

We chose pressure as a measure of discomfort based on the concept of the pain-pressure threshold (Belda-Lois et al., 2008), a pressure level that causes pain even on short duration of application. The exact value of this threshold varies significantly across body sections and population groups, and can cause pain at significantly lower pressures when applied for longer durations [reported as much as 50% lower by (Belda-Lois et al., 2008)].

**Figure 7:**
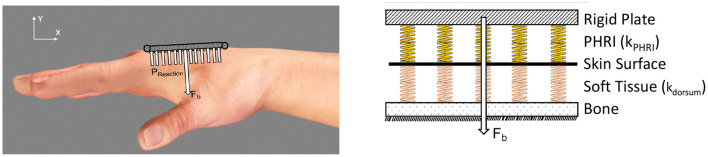
**(Left)** Hand dorsum and simplified PHRII with uniform stiffness, bias force (*F*_*b*_), applied using straps and approximated as a point load at the center, and reaction pressure (*P*_*reaction*_). **(Right)** Modeling all compliant elements between the human reference structure (our skeleton) and the rigid links of the robot. The stiffness of the hand dorsum (*k*_*dorsum*_ indicating the orange springs) and the PHRII (*k*_*pHRI*_ indicating the yellow springs) behave as a set of viscoelastic springs in series.

We model the PHRII (Figure 7 **Left**) as a discrete array of springs representing the hand dorsum (*k*_*dorsum*_) and the PHRII (*k*_*pHRI*_), respectively. These two arrays of springs are in series with each other (Figure 7 **Right**). This system is loaded with a constant bias force normal to the surface representing a generalized net force from one or more straps. External loading is resolved into the generalized net force and moment vectors, explained in more detail in section 2.3.1. Through analysis of the simplified system under external load, we deduce the shape of the desired effective stiffness (*k*_*eff*_) of this series spring model that allows us to minimize the peak pressures at the contact surface (Section 2.3.1). Using a numerical simulation environment, we compute the optimal effective stiffness gradient which satisfies the deduced shape profile from the analysis, and that also minimizes peak pressure (Section 2.3.2). Next, we quantify the stiffness profile of the experimenter's hand (*k*_*dorsum*_) through an indentation experiment with a robot (Section 2.3.3). The *k*_*eff*_ computed from the numerical simulation and *k*_*dorsum*_, obtained experimentally, are then used to compute the *k*_*pHRI*_, the optimal stiffness of the PHRII (Section 2.3.4).

#### 2.3.1. Analytical Determination of Optimal Stiffness Profile for the PHRII

**Figure 8:**
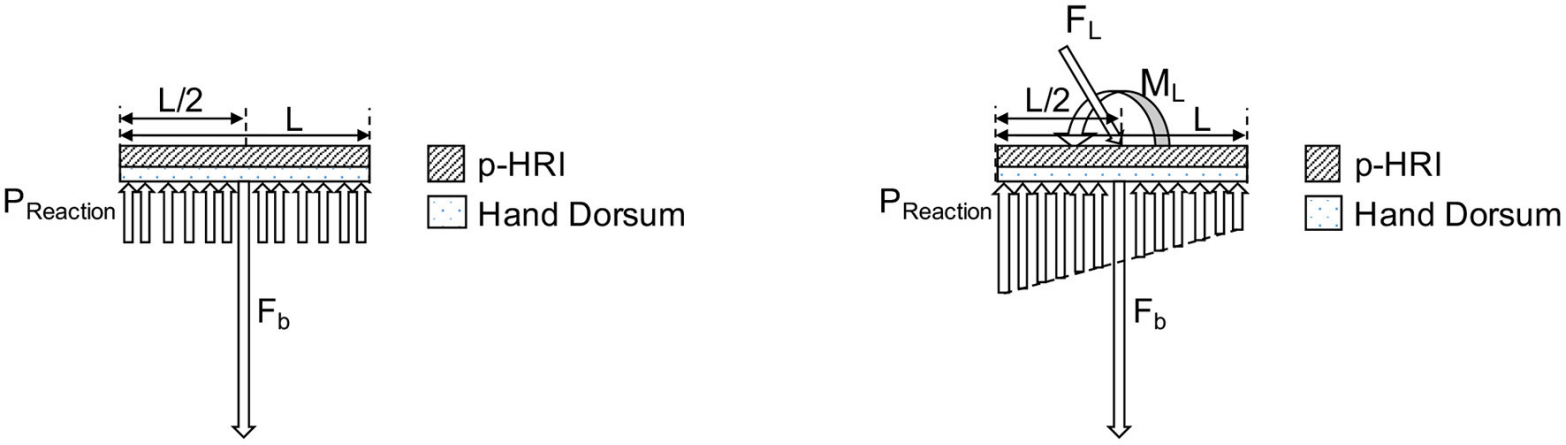
Model of hand dorsum and PHRII with uniform stiffness, bias force (*F*_*b*_), and uniform reaction pressure (*P*_*reaction*_) distribution.

To design an optimal PHRII for the hand dorsum, we simplify the complex interaction at the interface as two plates of length L and uniform width sandwiched between the rigid reference plate of the Maestro robot, and the rigid human bone. The robot reference plate is held to the dorsum by a bias force mimicking a strap (*F*_*b*_), applied normally and at L/2 as seen in Figure 8 **(Left)**.

We then load the PHRII with an external force mimicking the reaction forces from the Maestro actuators. Using the principle of transmissibility, we express the applied external force to the attachment plate as a combination of an equivalent force and moment applied at the center of the plate, placed coaxially with the bias force. The resulting reaction pressure (*P*_*reaction*_) distribution balances the net force (*F*_*b*_ plus the normal component of *F*_*L*_) and the external moment (*M*_*L*_) [Figure 8 **(Right)**].

Our objective is to balance the applied force and moment while minimizing peak reaction pressure (*C*_*i*_) along the contact surface between the two plates.


(1) $$C_{i}=max\left(P_{reaction}\right)$$

**Figure 9:**
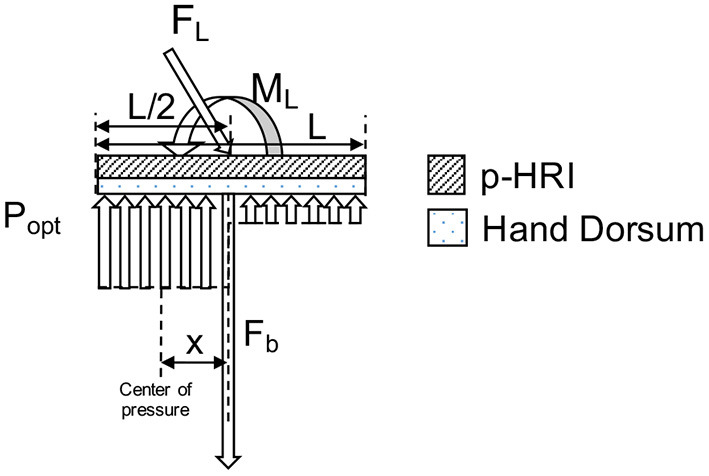
Model of hand dorsum and PHRII with minimized pressure distribution, bias force (*F*_*b*_), externally applied force (*F*_*L*_), moment load (*M*_*L*_) and resulting two regions of optimal reaction pressure (*P*_*opt*_) distribution.

Under the counter-clockwise external moment, *M*_*L*_, applied on the system, minimizing the cost function, *C*_*i*_ gives us a pressure distribution with two regions of optimized uniform reaction pressure (*P*_*opt*_) below the plate (Figure 9).

Since we assume that the sum of pressure is equal to the applied load, minimizing the peak pressure would distribute it over a larger area giving us a region of uniform pressure. However, uniform pressure across the entire plate cannot balance the counter-clockwise applied moment, which explains the existence of two sections of different uniform pressure. The direction of this reaction pressure distribution (seen in Figure 9) depends on the direction of the external loading and reverses itself when the external loading is in the clockwise direction.

The boundaries of the regions of uniform pressure shift depending on the ratio of external applied moment (*M*_*L*_) to the external applied force (*F*_*L*_). The position of “*x*,” the center of the highest pressure region in the optimal distribution (*P*_*opt*_), and “*P*_*peak*_,” the magnitude of the highest pressure between the two uniform distributions, are computed in Equations (2) and (3).


(2) $$x=\frac{M_{L}}{F_{L}+F_{b}}$$

(3) $$P_{peak}=\frac{F_{L}+F_{b}}{L-2x}$$

The optimal value of bias force, *F*_*b*_ of the attachment against the dorsum, to minimize *C*_*i*_ for the given force and moment loading configuration, is calculated to be the lowest value that gives us a non-negative pressure region (4):


(4) $$F_{b}=\frac{4M_{L}}{L}-F_{L}$$

By analyzing the possible solutions to achieve this optimal pressure, (*P*_*opt*_), a hyperbolic distribution with high stiffness in the center of the plate which tapers off toward the edge in the direction of the applied moment, *M*_*L*_, is a solution to the equation, though its exact parameters are yet to be computed. We approximate this hyperbolic representation to a linear stiffness profile for our experiment, decreasing from the highest stiffness at the middle of the attachment (*k*_*mid*_), and symmetrically tapering off to a minimum at each edge (*k*_*edge*_). The symmetry allows the resulting profile to hold true for external moment loads in either direction. To obtain *k*_*pHRI*_, we need to obtain *k*_*dorsum*_ in addition to knowing *k*_*eff*_.

#### 2.3.2. Numerical Computation of the Desired Stiffness Profile

We used the HuRoSim environment to compute the effective spatial stiffness gradient that minimizes peak pressure. The environment was also used to characterize the relationship between the bias force, the gradient of stiffness, relative displacement between the robot and the human, and the peak pressure over the hand dorsum.

For this experiment, the dorsum surface was discretized into 15 total points, with the PHRII interacting with the underlying human metacarpal through the stiffnesses *k*_*pHRI*_ and *k*_*dorsum*_ in series at each point. Piece-wise linear values for both stiffness were used, with *k*_*dorsum*_ taken from our experimental results. The system was simulated for varying applied force (*F*_*L*_) and moment loads (*M*_*L*_) with varying *k*_*pHRI*_ profiles to examine the resulting pressure distribution. The relative displacement of the PHRII with respect to the underlying bone, due to *F*_*L*_, was also captured for each stiffness profile, and these results are presented in the next section.

#### 2.3.3. Quantifying Tissue Response to Indentation

Tissue stiffness on the human body is quantified by applying force over a range of displacement.

**Figure 10:**
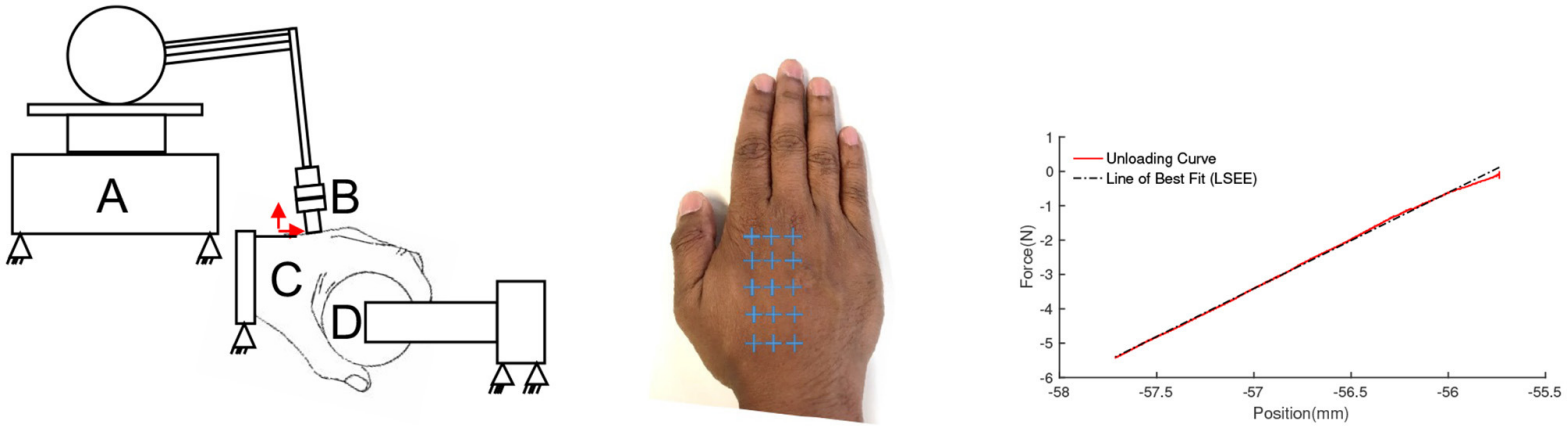
**(Left)** Phantom premium 1.5 high force haptic renderer **(A)**, instrumented with an ATI nano 17 6-axis force torque transducer **(B)**, probing the hand dorsum **(C)** over a spatial grid while the subject grasps a spherical object instrumented with an ATI Nano 17 **(D)**. **(Middle)** Locations of measured stiffness measured over the 2nd & 3rd metacarpal, and 2nd-3rd intermetacarpal region. The blue “+” symbols represent the sites of indentation on the hand dorsum. **(Right)** Fitting a line to the force deflection curve from indentation experiments. The slope of the fitted line corresponds to the measured stiffness.

To measure *k*_*dorsum*_, we designed an indentation system comprised of a Phantom Premium 1.5 high force haptic renderer, which has a high positional accuracy of the end effector (7*10^−6^m). This was used along with an ATI Nano 17 force torque transducer (having a high force torque sensing accuracy of 0.001N) attached at the end of the linkage as an indenter to probe the hand dorsum (Figure 10). We selected five points along a line between the metacarpophalangeal joint and the radial styloid process along the 2^*nd*^ metacarpal bone, the 3^*rd*^ metacarpal bone, and along a line between the two metacarpal bones, in the inter-metacarpal region. This region was selected to correspond to the area of the attachment plate on the Maestro exoskeleton. Figure 10 **(Middle)** shows the regions selected for indentation.

The wrist and arm were supported in braces, and a consistent grasp object was used across trials to minimize its influence on stiffness distributions due to the changes in bone locations and muscle recruitment strategy, as seen in Figure 10 **(Left)**. The wrist and arm supports were positioned to level the hand dorsum in the transverse anatomical plane. The phantom probe is then manually led once to each point marked on the dorsum for indentation. The probe uses these points as input into an interpolator to compute a spatial trajectory to follow. The phantom is driven in an open-loop position controlled configuration.


(5) $$k_{dorsum}=\frac{k_{measured}*k_{indenter}}{k_{indenter}-k_{measured}}$$

Quantifying the stiffness of the indentation system in the direction normal to the hand dorsum demonstrated the need to account for this value in estimating the stiffness of the hand dorsum. The stiffness of the indentation system (*k*_*indenter*_) was found to be 2.67 N/mm along the workspace. We account for this stiffness in the measurement of the hand dorsum stiffness (*k*_*dorsum*_) by modeling the interaction between the indentation system and the hand dorsum as two springs in series. The measured stiffness of the hand dorsum, *k*_*measured*_, (Figure 10 **Right**) is used along with *k*_*indenter*_ to calculate the *k*_*dorsum*_ Equation (5).

We made five sequential repeated measures to estimate the variance in the measured stiffness at each point. The observed variance was attributed to movement in the hand. The hand dorsum stiffness data is collected from one pilot subject only, and with a probe having a square base with 4*mm* edges and 1.5*mm* filets on each edge to minimize discomfort during indentation. The indenter profile and size were chosen iteratively based on the relative distribution of the hard and soft tissue structures in the hand. Increasing the resolution of the grid beyond the current levels introduced errors due to partial overlap of soft and hard tissue at the points of measurement.

#### 2.3.4. Calculating the Optimized Padding Stiffness

In our model, *k*_*eff*_ between the human bone and the Maestro robot's reference plate is comprised of *k*_*dorsum*_ and *k*_*pHRI*_ in series. Therefore, once we have numerically computed *k*_*eff*_, and measured *k*_*dorsum*_ through the indentation experiment, the required *k*_*pHRI*_ can be calculated at every point on the attachment surface Equation (6). This gives us a PHRII stiffness profile that should generate the minimum peak pressure, or the optimal pressure profile (*P*_*opt*_) on the hand dorsum for the given *F*_*L*_ and *M*_*L*_.


(6) $$k_{pHRI}=\frac{k_{eff}*k_{dorsum}}{k_{dorsum}-k_{eff}}$$

## 3. Results

### 3.1. Experiment 1: Predicting Physical Interaction Using HuRoSim

HuRoSim offered many insights into the interaction between the exoskeleton and the hand at the PHRII. Specifically, the simulation environment was able to predict the directions and magnitude of displacements and forces that one would expect on the PHRII. The following sections describe some of the observations predicted by HuRoSim that were also confirmed by empirical experiments with the Maestro hand exoskeleton under identical conditions.

#### 3.1.1. The Effects of Strap Pretension and Applied Moment Load

**Figure 11:**
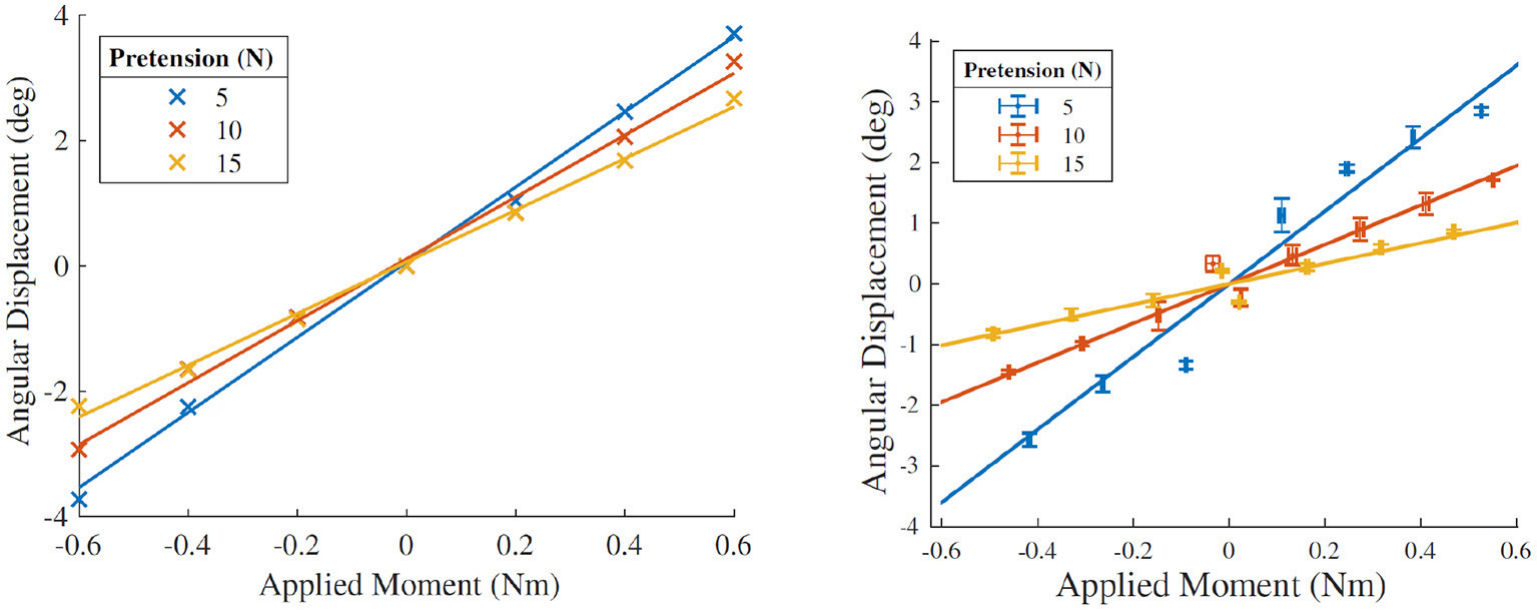
**(Left)** Simulated angular displacement of the rigid surface of the dorsum attachment on application of moment loading under different conditions of strap stiffness. **(Right)** Angular displacement observed under identical conditions in the physical experiment. The HuRoSim model is able to predict the trends in effect of both the varied parameters of strap pretension and moment loading. However, under higher pretension, where the non-linear behavior of human soft tissue becomes more pronounced, there is expected divergence between the predicted and experimental values.

The effect of varying the applied moment loading and the strap pretension were studied in both the HuRoSim environment, as well as in the physical experiment. Figure 11 **(Left)** shows the angular displacement of the exoskeleton dorsum attachment on application of moment load by the exoskeleton about the MCP joint. HuRoSim predicted an angular displacement that increased approximately proportional to the applied moment load, and that was reduced on increased strap pretension. Physical experiments under the same conditions confirmed both trends as seen in Figure 11 **(Right)**. The experimental values of angular displacement under low strap pretension matched the predicted values quite closely. However, under higher pretension, where the non-linear behavior of human soft tissue becomes more pronounced, there is expected divergence between the predicted and experimental values.

In addition, when the exoskeleton applies an extension torque about the MCP joint, HuRoSim also predicted an increase in pressure at the location on the PHRII on the hand dorsum closer to the joint while the location further away from the MCP joint experiences a reduction in pressure. The inverse is true when the actuator applies a flexion torque about the MCP joint. HuRoSim indicated that the magnitude of these increases with respect to the applied moment was found to be dependent on the length of the exoskeleton attachment and on the geometry of the PHRII at the contact locations on the hand dorsum.

#### 3.1.2. Liftoff From the Finger Surface

**Figure 12:**
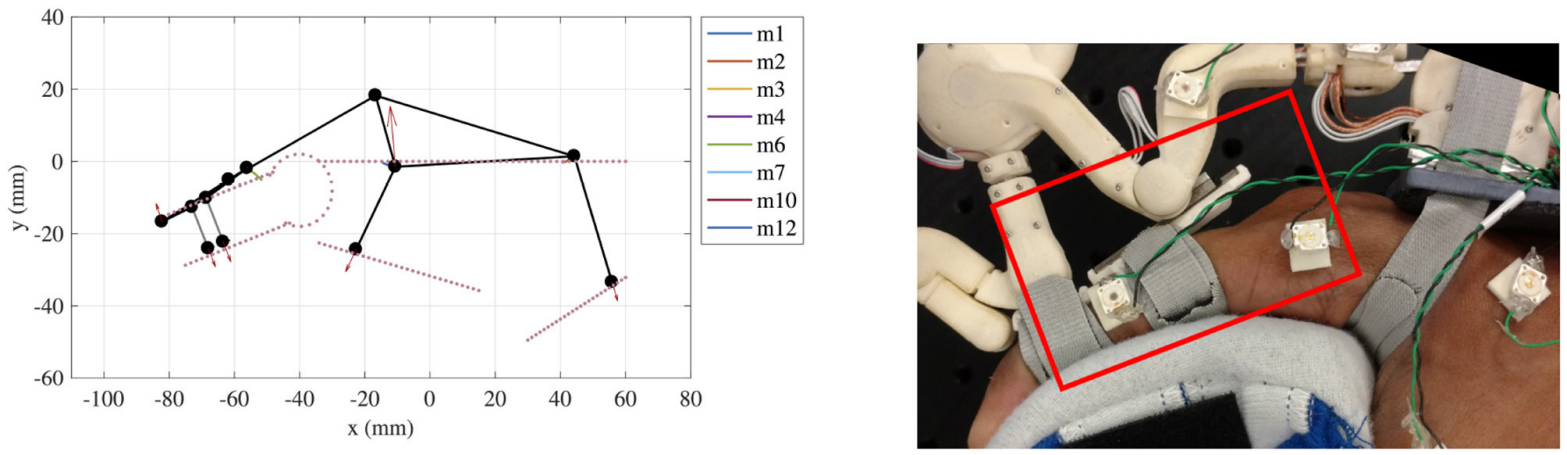
**(Left)** An example of simulated prediction of liftoff at the proximal phalanx attachment. **(Right)** The physical manifestation of the same during experimental characterization.

As a consequence of the distribution of pressures described above, HuRoSim predicted that the end of the PHRII closest to the MCP joint would lift off and lose contact with the skin surface on the application of extension torques by the exoskeleton. When we replicated the simulation empirically with the Maestro hand exoskeleton, we observed the lift off phenomenon predicted by HuRoSim (Figure 12).

#### 3.1.3. Effect of Varying Strap Stiffness

The effect of varying strap stiffness was studied using the simulation environment to identify the optimal strap stiffness to minimize both output metrics (relative displacement and forces across the interface). HuRoSim's initial results indicated that the best performance on both metrics occurred with effectively inelastic straps, or straps with extremely high stiffness.

This result was unexpected since pilot user feedback with the maestro exoskeleton reported that mild elasticity was the most comfortable. Exploring different variables in the system, it was observed that in the actual human hand, co-contraction of muscles in response to the applied load leads to an increase in the thickness of the muscle body as it is flexed, and resulting changes in the cross sectional thickness of the hand. These also vary depending on the musculature of the individual subject and the magnitude of voluntary contraction. When the simulation was tested with approximate values of the cross section variation, a slightly reduced strap stiffness was found to produce lower pressure distributions with minimal trade-off in the relative displacement values.

The values of this optimal reduced stiffness, however, are dependent on the magnitude of cross section increase due to co-contraction. Future work can perform the simulation using magnitudes of cross sectional variation sourced from literature or experiment to inform design values of this optimal stiffness.

### 3.2. Experiment 2: Optimizing the Design of the Hand Dorsum PHRII

**Figure 13:**
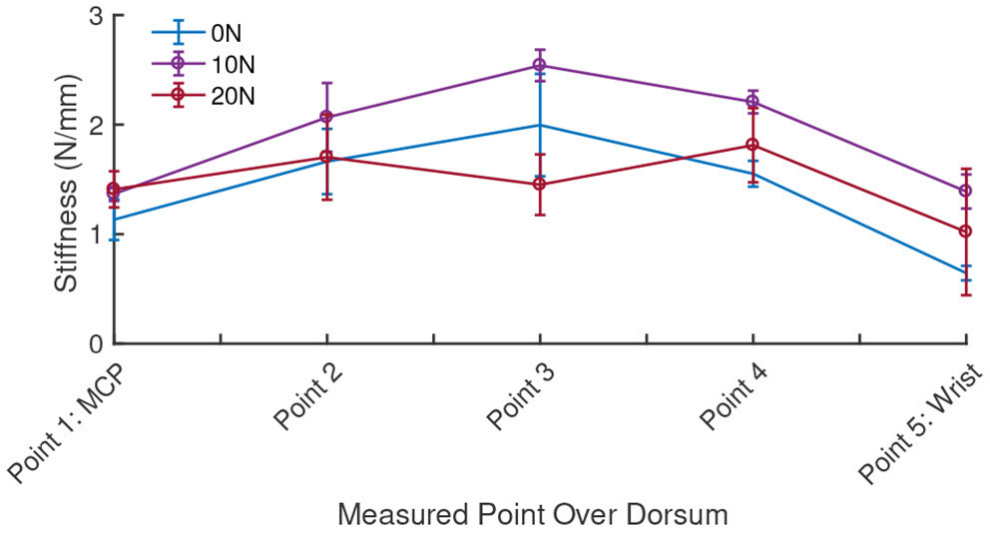
Measured stiffness of the hand dorsum for five equally spaced points along the second metacarpal at three levels of grasp force applied by the subject.

The stiffness of the hand dorsum was measured and characterized with five repetitions over each of the 15 chosen points, distributed equally over the 2nd metacarpal, 3rd metacarpal and the inter-metacarpal gap between these bones on a single subject's hand (Figure 13). On average, *k*_*dorsum*_ was measured to be 1.0876 ± 0.40 N/mm over a range of from 0.54 to 1.59 N/mm. The region of the dorsum above the metacarpal bones was found to be stiffer (1.1285 ± 0.43 N/mm) than the region between the bones (1.0060 ± 0.36 N/mm) that accommodate soft tissue. Increasing force of grasp led to an increase in measured dorsum stiffness (Figure 13), however, the PHRII stiffness computed here is for a grasp force of 0 N.

**Figure 14:**
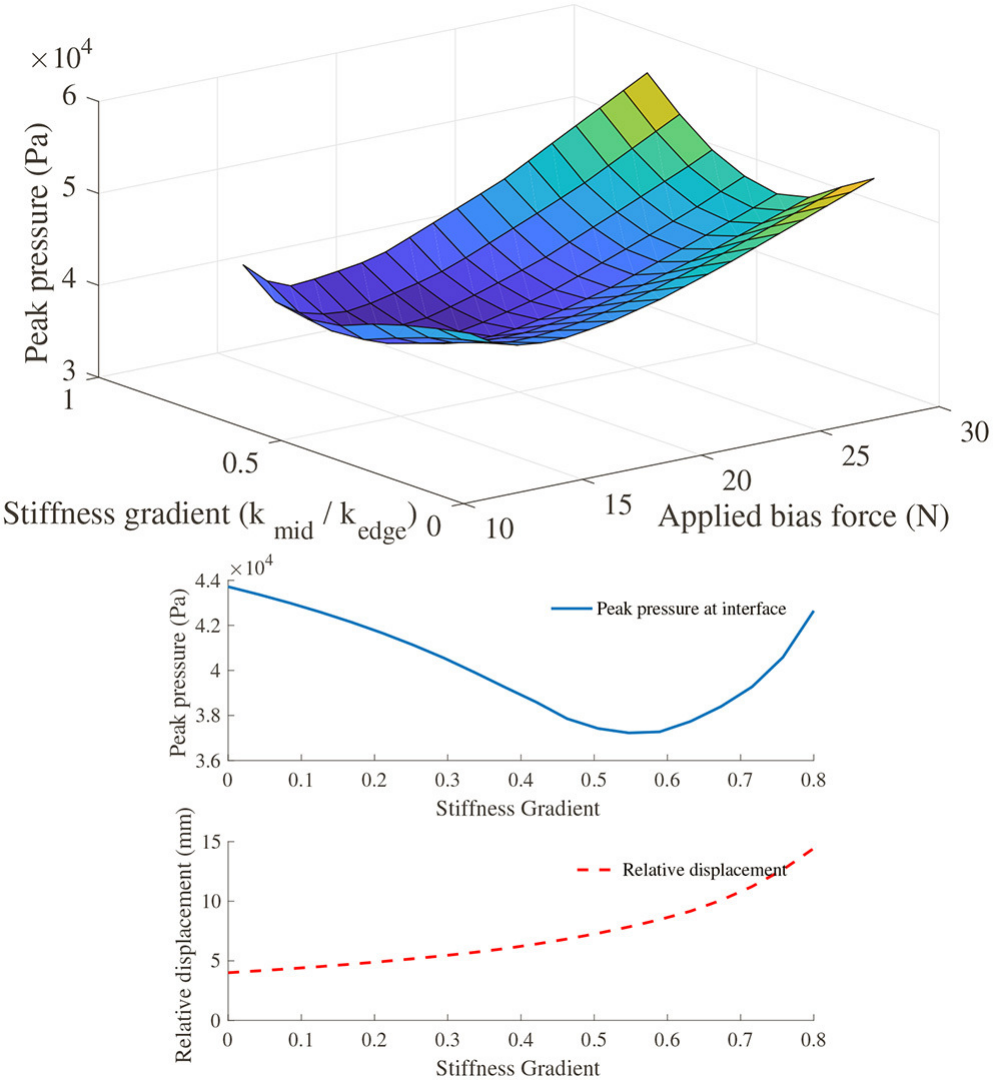
**(Top)** Surface plot showing the effect of varying bias force and stiffness profiles on the peak pressure (as a measure of user comfort) across the PHRII on the hand dorsum. **(Bottom)** Trade-off between peak pressure (as a measure of user comfort) and relative displacement on varying the stiffness profile gradient.

For our optimization experiment, the key metrics of performance considered were the peak pressure over the dorsum, and the relative displacement of the exoskeleton with respect to the hand. The system was simulated across a range of applied moment, bias force and stiffness gradient to characterize the effects of these variables on our performance metrics. Figure 14 shows a surface of the combined effect on peak pressure at the dorsum interface due to changes in bias force and stiffness profiles, while holding the applied moment constant. It shows that the minimum peak pressure is achieved at the bias load as calculated in Equation (4) for all stiffness profiles. When viewing the effect of varying stiffness profile gradients, we again observe a local minimum in the peak pressure at the dorsum, which is explained in more detail below.

**Figure 15:**
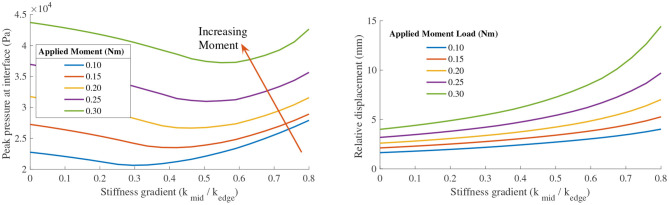
**(Left)** Plot of peak pressure at the PHRII interface (as a measure of user comfort) vs the stiffness profile gradient for different applied moment loads at constant bias force. **(Right)** Plot of the effect on relative displacement between the human skeleton and the attachment base on changing the stiffness profile gradient of the PHRII.

Looking at stiffness profile, the highest values of peak pressure are observed at the highest applied moment load for all effective stiffness profiles. However, for any given fixed applied moment, we observe an improvement in peak pressure at the dorsum when the effective stiffness between the human bone and the robot is varied in a gradient from the center to the edge of the interface, as seen in Figure 15 **(Left)**. The percentage improvement increases with increasing applied moment for the same value of bias force. Additionally, the optimal value of stiffness profile gradient is seen to increase with the applied moment. This corroborates well with our analytical prediction of the a load dependent optimal value of stiffness gradient.

When considering the effect of these same variables on the relative displacement, it becomes evident that we have a trade-off between our performance metrics of comfort and relative displacement. Figure 15 **(Right)** shows the effect on relative displacement due to a change in stiffness profiles with the applied force and moment loading held constant for each of the plotted lines. Contrary to the effect on peak pressure shown earlier, the relative displacement increases with the stiffness profile gradient.

This is more clearly illustrated in Figure 14, where the two output metrics are plotted against a common *x*-axis of the stiffness profile gradient. It shows that increasing the stiffness profile gradient to improve the peak pressure across the interface has an adverse effect on the relative displacement. Thus, a practical design would be one in which we choose a trade-off between the two desired outcomes and pick our stiffness gradient based on an optimal weighting between the two.

## 4. Discussion

### 4.1. Simulation Insights

We presented a novel method to optimize the design of the PHRII between a wearable exoskeleton and the human hand. By characterizing the mechanical properties of tissues at a few locations on the hand dorsum, we demonstrated how limited experimental data can be used to optimize the design of the PHRII through the use of a custom simulation environment, HuRoSim.

For this simulation, input parameters of interest were strap pre-tension and the applied moment load. Important simulation output parameters were the relative displacement between the exoskeleton and the human hand, and human comfort (measured as peak pressure across the dorsum surface).

Relative displacement was found to increase non-linearly with increasing applied moment. Relative displacement was similarly found to decrease with increasing strap pre-tension. This relation is expected since almost all of the stiffness in the human system, as well as the padding and straps are non-linear and increase with increasing strain. It is important to note that the magnitudes and directions of the above effects are configuration dependent and can change drastically with different link lengths of the robot exoskeleton, MCP joint angle, and other joints in the system as well. These dependencies, however, are very easily accepted as inputs to the numerical simulation model if we wish to study them independently.

These results are the first of its kind for the hand. The trends observed here for this PHRII study agree with results observed by other experiments conducted on the lower limbs (Quinlivan et al., 2015).

### 4.2. Experiment 1: Predicting Physical Interaction Using HuRoSim

Experimental characterization of the identical system as simulated was carried out to confirm the behavior of the simulated system in a physical setting. This experimental setup is a highly instrumented version of an actual human-robot system specifically to study the effects of force across the PHRII interface. The results gained from this experimentation show the level of experimental characterization required to achieve similar insights that a simulation approach makes possible. This simulation approach could be advantageous in both cost and time for the iterative stages of design refinement during a coupled human-robot system's design lifecycle.

Future work of simulation models could be to develop bounds on a range of slip along the skin surface, since this movement is an important part of the PHRII sstem.

By producing outcomes that align with the experimental results, this experiment demonstrates that HuRoSim is capable of predicting phyisiologically observed behavior with only estimated information of the individual components of the system. Further study will definitely benefit from more detailed experimental characterization, accounting for anthropometric variations. This could further improve simulation accuracy.

### 4.3. Experiment 2: Optimizing the Design of the Hand Dorsum PHRII

We used a simulation based approach to assess the pressure distribution that would be generated across a single PHRII due to the application of force and moment loading. Our simulation of a simple PHRII system involving just a single interaction surface shows that it is possible to identify optimal values of parameters such as the strap bias load, and the padding stiffness profile. This is an important result that can be used in the design of more ergonomic and effective PHRIIs for coupled human robot systems.

Validation of the existence of these optimal PHRI parameters was carried out using analytical calculations of the test cases. While experimental characterization should be done as well in our future work, these analytical calculations of our system provided us with a theoretical explanation of the optimal values that were numerically identified by simulation. In the case of the optimal bias force for a given applied moment, we were also able to obtain a predicted optimal value from the analytical calculation, and this matched well with the simulation results.

We also identified the tradeoff between the peak pressure at the interface as a measure of user comfort, and the relative displacement across the interface which is a measure of position error in the system. Parameters that produce such tradeoffs in optimal pressure distributions include the stiffness profile gradient and the bias force of the PHRII. Knowledge of this tradeoff from simulation outputs allows us to make informed design choices when choosing the parameters of our PHRIIs.

We would recommend simulating a PHRII during its design phase in order to better choose its properties at the onset rather than having to go through theoretical iterations. While a lot of the optimal parameters do depend on the applied loads and geometry of the system, our analysis does bring out one general recommendation as well. Any PHRI that is to be subjected to high moment loading should ideally be designed with stiffer regions near its center, and regions of low stiffness close to its edges.

The design of such custom designed properties across a PHRII surface has already been successfully shown by Petron et al. (2016) in their design of the variable impedance socket for transtibial amputees. The work presented in this paper expands on the idea by using a more generalized setting of a single PHRII rather than a closed socket, and also focuses on cases that involve a high application of moment loads. We propose that by the use of our simulation based approach, effects of different parameters of the PHRII and the applied loads can be effectively characterized and used to drive design choices.

HuRoSim's simulation engine was custom built on Matlab (Mathworks Inc., MA) to have a greater control over the design of the dynamic solver. Future versions of this environment can be implemented on commercial multi-body physics platforms to build a real-time system capable of solving continuous dynamics of the system. The experimental characterization of the tissue properties was quasi-static and can be extended to quantify dynamic response of the tissue to loading. Modeling the tissue response in greater detail can enable the design of PHRII optimized for dynamic loads applied during task performance.

HuRoSim's modular construction enables it to not be limited to applications of just the hand, but to any human-robot coupling where both comfort and positional accuracy are of importance. Other upper extremety examples such as supernumary fingers (Hussain et al., 2017; Salvietti et al., 2017) and lower extremity examples such as ankle-foot orthoses (Kim et al., 2020). Simulation based design could potentially be of significant use for such applications.

## 5. Conclusion

In this paper, we presented a novel systematic approach to quantifying the complex interactions at the Physical Human Robot Interaction Interface (PHRII) for wearable robots coupled to the body. We demonstrated how a novel simulation environment (HuRoSim) can be utilized with experimental data to improve the design of the PHRII for optimal comfort and performance. By utilizing this approach, design loops can leverage the power of limited experimental measurements that inform fast and inexpensive simulation tools to generate design recommendations.

Our approach fills an important gap in the literature between difficult and error-prone experimental characterization of the behavior of the pHRII, and expensive iterative physical prototyping for the design of the interface between the human body and the device.

We demonstrate that reduced order models informed by limited experimental measurements of human tissue provide sufficient information to optimize PHRII design. This work represents the beginning of a step change in the design of PHRII for robots attached to the body. It demonstrates how complex physical interactions between man and machine can be simplified using first principles to generate actionable recommendations for reduced design time and complexity.

## Data Availability Statement

The raw data supporting the conclusions of this article will be made available by the authors, without undue reservation.

## Ethics Statement

The studies involving human participants were reviewed and approved by Institutional Review Board, University of Texas at Austin. Study number: 2013-05-0126. The patients/participants provided their written informed consent to participate in this study.

## Author Contributions

RJ contributed to the development of the simulation engine of the platform under the guidance of AD. GM contributed to experimental acquisition of the hand dorsum properties. RJ and GM contributed to analysis of the data from simulation and physical experiments. All authors contributed to writing, reviewing, and proofreading the manuscript.

## Conflict of Interest

RJ was employed by the company Harmonic Bionics Inc. The remaining authors declare that the research was conducted in the absence of any commercial or financial relationships that could be construed as a potential conflict of interest.

## Publisher's Note

All claims expressed in this article are solely those of the authors and do not necessarily represent those of their affiliated organizations, or those of the publisher, the editors and the reviewers. Any product that may be evaluated in this article, or claim that may be made by its manufacturer, is not guaranteed or endorsed by the publisher.
